# Cardiovascular Risk Behavior among Sedentary Female Smokers and Smoking Cessation Outcomes

**DOI:** 10.1186/1617-9625-3-1-7

**Published:** 2005-12-15

**Authors:** Tellervo Korhonen, Taru Kinnunen, Zandra Quiles, Robert F Leeman, Donna Medaglia Terwal, Arthur J Garvey

**Affiliations:** 1 Tobacco Dependence Treatment and Research, Harvard School of Dental Medicine, Boston, MA; 2 Lehman College – CUNY, New York, NY; 3 Department of Psychiatry, Yale University School of Medicine, New Haven, CT

## Abstract

**Background:**

We examined female sedentary smokers' additional cardiovascular disease (CVD) risk behaviors and their associations to smoking cessation.

**Methods:**

This study was part of a randomized controlled trial testing the effectiveness of exercise and nicotine gum in smoking cessation. Included in the analyses were 148 participants. Dietary habits and alcohol consumption were measured as additional CVD risk behaviors. High-fat diet and heavy alcohol use were considered those risk behaviors. Nicotine dependence, length of the longest quit attempt, depressive symptoms, self-efficacy, and education were examined as other baseline variables. Abstinence from tobacco was recorded through 12 months.

**Results:**

Diet was related to depressive symptoms at baseline. Alcohol use was related to nicotine dependence and education level. Heavy alcohol use alone and accumulation of two added risk behaviors predicted poorer smoking cessation outcome. Although diet alone was not associated with cessation outcome the high-fat diet interacted with depressive symptoms, such that the depressed women with high-fat diet were significantly more likely to relapse in their quit attempt compared to other subgroups.

**Conclusion:**

Non-moderate alcohol use alone and accumulation of multiple CVD risk behaviors seem to be associated with lower success in smoking cessation.

## Introduction

Smoking cessation results in many positive health consequences; most immediately and substantially cessation reduces the risk for coronary heart disease and other cardiovascular diseases (CVD) [1]. It has been suggested that treating tobacco dependence is more cost-effective than any other preventive cardiology measure [2]. However, many clinical trial studies report declining abstinence rates over time from the 1970s to 1990s [3,4]. One explanation is that nowadays smokers tend to be highly dependent on nicotine [5]. Highly dependent smokers tend to have lower education, more depressive symptoms, consume unhealthy foods, and report more hazardous drinking and less physical activity than smokers with low dependence [6-9].

Studies in the U.S. have shown that smoking women are more likely to use alcohol, marijuana and cocaine, and have greater substance use severity than the non-smokers [10-13]. Further, smoking is predictive of many other negative health behaviors. Indeed, while unhealthy behaviors have shown pair-wise and multiple accumulation, smoking in particular, has the strongest and most consistent associations with other unhealthy behaviors [8,14-16]. This clustering of smoking and other unhealthy behaviors, "risk behavior syndrome", may have already emerged at a young age, such as during elementary school [17,18]. Readiness to quit smoking is also related to other health behaviors. Smokers who do not want to quit smoking (consonant smokers) differ in their health behavior from those willing to quit (dissonant smokers). Smokers in the pre-contemplation stage, not planning to quit within the next six months, demonstrate fewer positive health practices [19]. Particularly, female consonant smokers tend to be more sedentary and heavier drinkers than female dissonant smokers [20].

The cardiovascular association of other unfavorable health behaviors besides smoking, such as alcohol use and diet, has been documented [21-23]. Alcohol use has a J-shape association to risk of CVD suggesting that moderate alcohol consumption would be protective whereas no alcohol drinking and heavy alcohol use would be risk factors [23]. There is, however, a recent study suggesting that this J-shape association would not be true among current smokers [24]. In relation to CVD, the unhealthy dietary practices include the high consumption of saturated fats, salt and refined carbohydrates, as well as low consumption of fruits and vegetables – and these tend to cluster together [21,22,25]. Further, smoking in combination with other risk factors has a particularly high impact on total CVD risk [26].

Although the presence of multiple health risk behaviors is related to predictors of continued smoking, such as nicotine dependence, the association of those unfavorable health behaviors with smoking cessation outcomes is still unclear. Particularly for female smokers, dietary intake plays an important role in smoking cessation via post-cessation weight gain [1]. In addition, smokers are less likely to be ready to make positive changes in their dietary fat and fiber intakes [27]. Based on these notions it would be relevant to argue that a smoker with an unhealthy diet would be more likely to gain weight during quitting and thus, more likely to relapse because of weight concerns. There is only one earlier study examining prospective relationship between diet and smoking cessation outcomes [28]. In this particular study the unhealthy diet was analyzed together with low physical activity level. The study found a significant association between high dietary fat intake/low physical activity level and smoking dependency among men and women. However, the association to cessation outcome was significant only among men but not among women. Shiffman et al. [29] have suggested that drinking alcohol in general is a significant precipitant of smoking relapse after a quit attempt. However, no previous study has examined whether the association of alcohol use with smoking cessation outcome would follow similar J-shape found in relation to CVD [23] – recently challenged by specific findings among smokers [24].

To understand the complex reasons for women's difficulties in smoking cessation there is need for a more comprehensive analyses of potential risk factors of high relapse rate after a quit attempt. It is known that various health behaviors, in addition to smoking, carry a significant risk for CVD. However, it is still unclear, if and how those behaviors would be risk factors for smoking cessation. Our main objective was to analyze if female sedentary smokers' additional CVD health risk behaviors, diet and alcohol use, predict abstinence from tobacco use. The secondary objective was to examine these risk behaviors' association to other variables related to smoking cessation. These include variables which reflect smoking-related (nicotine dependence, length of the longest past quit attempt), socio-economic (education), and psycho-social (depressive symptoms, self-efficacy) components of the smoking cessation process.

## Methods

### Study design

Sedentary female smokers from the Greater Boston area were recruited for a randomized controlled trial testing the effectiveness of aerobic exercise as an adjunct to nicotine gum therapy. The inclusion criteria included an age of 18–55 years[JS1], no major cardiac conditions (e.g. history of acute cardiac events including MI or current abnormal resting EKG), not pregnant or planning pregnancy, not suffering from any severe psychiatric conditions, bleeding ulcers or insulin dependent diabetes.

The participants were followed from 3 weeks before cessation to 1 year post-cessation. We provided all participants with nicotine gum treatment and brief counseling (how to use gum, possible withdrawal symptoms and how to deal with them). We randomly assigned the participants to one of three conditions: (1) Exercise intervention condition ('exercise group'). This consisted of two 45-minute exercise sessions per week from 3 weeks pre-cessation through 2 weeks post-cessation. At that time, and continuing through 16 weeks post-cessation, exercise sessions were reduced to one per week. In addition, the participants were asked to exercise 2–3 times per week on their own. (2) Equal contact control condition ('wellness group'). This included wellness lectures and discussions for the same time period and with the same frequency and duration as the exercise intervention. Compliance with both 'exercise group' and 'wellness group' was monitored through 16 weeks by recording attendance of the sessions in both groups plus by self-reported frequency of home exercise in the exercise group. (3) Standard care control condition ('control group').[JS2] This included only the nicotine gum treatment and brief counseling received by the other two groups as well. The present set of analyses is not investigating the intervention effect, and only those conditions that had equal contact times and similar abstinence rates (the main outcome measure of the trial), were included in the present report. The sample consisted of 148 participants randomized into the 'exercise group' (n = 92) and 'wellness group' (n = 56). Cox Proportional Hazard model Survival analysis for 12-month follow up indicated no significant difference in abstinence rates between the exercise and wellness group (*p *= 0.600). The standard care 'control group' was excluded as it differed in the main outcome both from the 'wellness group' and 'exercise group' (p < .05) and did not have equal contact.

**Figure 1:**
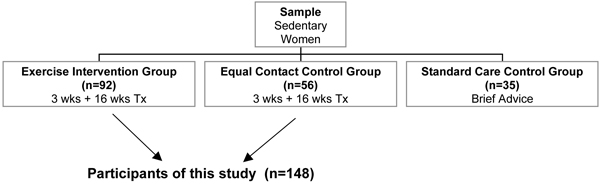
**Experimental design of the trial**.

Figure 1 shows the experimental design of the trial with numbers of participants in each group. The research study was approved by the Harvard Medical School Office for Research Subject Protection and by the Brigham and Women's Hospital's Human Research Committee. All participants signed the Informed Consent form.

### Participants

Participant characteristics are shown in Table 1. On average, participants were 38.1 (SD = 9.6) years old and smoking 18.2 (SD = 8.1) cigarettes per day.

**Table 1:** Characteristics of Study Participants at Baseline

	(%)	n
Race/ethnicity		
White	80.1	117
African American	12.3	18
Hispanic	4.8	7
Other	2.8	4
		
Marital status		
Single	56.5	83
Married	19.7	29
Divorced/separated/widowed	23.8	35
		
Education		
High school graduate or G.E.D.	8.1	12
Some college/business/technical school	46.0	68
College graduate	30.4	45
Post-college degree	15.5	23

	Mean	(SD)

Age	38.1	(9.6)
Number of cigarettes/day	18.3	(8.1)

The majority were Caucasian/white (80.1%) and single, separated, divorced or widowed (80.3%). All participants had at least high school education.

### Assessments

#### Risk behaviors

As mentioned earlier, all participants were daily smokers (>5 cigarettes per day) with a sedentary lifestyle (exercising <3 times per week for 30 minutes). Thus, every participant was engaged at least in two compromising behaviors, which have been identified as CVD risk factors [30,31]. As additional CVD risk behaviors, we measured dietary behavior and alcohol consumption [21-23].

#### Dietary behavior

In the baseline questionnaire we asked the participants how often they had eaten various foods during the past week, such as fruit, juice, green salad, cooked vegetables, hamburgers, hot dogs, sausages, french fries, potato chips, cookies, etc. The frequency for each type of food product was scored as follows: 0 = never, 1 = in 1–2 days, 2 = in 3–5 days, 3 = in 6–7 days, 4 = every day. We used this data and our own method to categorize the participants into three groups. The "high-fat" group was defined as a diet high in saturated fats found in animal products, such as meat, eggs and dairy. Thus, high frequency (scored as 3–4) of eating hamburgers, hot dogs, sweet pastries etc. and low frequency (scored as 0–1) of eating fruit, juice, green salad, cooked vegetables etc. was categorized as a "high-fat" diet. Respectively, high frequency of eating fruit, juice, green salad etc. and low frequency of eating hamburgers, hot dogs, sausages, etc. were categorized as a "high-vegetable" diet. Those who did not fit into these two categories were classified into the "mixed diet" category. The proportions of participants in each group were as follows: high-fat (27%), high-vegetable (30%), and mixed diet (43%). The "high-fat" diet was regarded as a dietary risk behavior, most of the foods in this group being high in saturated fats, salt, and refined carbohydrates, but low in fruits and vegetables [21,22].

#### Alcohol use

Data on alcohol consumption were based on questions of how many cans/bottles (12 oz) of beer, glasses of wine (6 oz) or mixed drinks (e.g. scotch, brandy, gin, vodka) our participants drink per week on average. Based on sum scores, we created three alcohol consumption groups as follows: no alcohol use at all (29% of the participants), 1–7 drinks per week (mean = 3.5, SD = 1.9) (49% of the participants), and more than seven drinks per week (mean = 14, SD = 7.1) (22% of the participants). The definition of specific risk behavior group was difficult because of inconsistent evidence. According to the J-shaped relationship of alcohol use and risk of CVD, non-use of alcohol on one hand, and more than 7 drinks per week on the other hand should be regarded as alcohol-related risk behavior for CVD [23], suggesting that the two first groups could be merged as one risk group. However, this J-shaped association has been recently challenged among smokers [24]. Thus, in relation to smoking cessation, we analyzed alcohol consumption in those three separate categories.

#### Other baseline variables

Nicotine dependence, length of the longest past quit attempt, depressive symptoms, self-efficacy, and education were examined as other baseline variables. These variables were selected to reflect smoking-related, psycho-social and socio-economic components of the smoking cessation process. Earlier studies have suggested their association with continuing smoking [32], smoking cessation [33-37], or other health behaviors [6,28]. Nicotine dependence was assessed with the Fagerström test for Nicotine Dependence (FTND) [38], a 6-item revision of the Fagerström Tolerance Questionnaire. Scores of the FTND range from 0 to 10. Length of the longest past quit attempt was based on the question "What is the longest time you have ever been able to quit smoking?" Participants were asked to record time by year, month, week, and/or days. For data analyses, we converted these reports into days of abstinence. To measure participants' depression status at baseline we used the Center for Epidemiological Studies Depression Scale (CES-D) [39]. The CES-D is an established self-report measure of both the frequency and severity of depressive symptoms. It consists of 20 symptoms that were rated on how frequently a person had experienced a given symptom during the past week. The appropriate items were reverse-scored and responses were summed to create a depression score, which can range from 0 to 60. In accordance with the standard scoring procedure for the CES-D, participants who scored higher than 15 were classified as depressed and those who scored 15 or below were classified as non-depressed [39]. Self-efficacy was measured with the item "How confident are you that you will be able to quit smoking for the next 3 months?" rated on a 5-point scale ranging from 0, very slightly or not at all, to 4, extremely confident. Education was measured as the highest level of formal education ranging from "Grade school" up to "Post-graduate degree".

#### Abstinence

Abstinence from cigarettes was measured immediately after quitting. The definition of relapse implemented in this study was taken from the recommendations of the National Task Force of Relapse (7 consecutive days or episodes of smoking)[40]. For this study abstinence was recorded at 3, 7, 14, 30, 60, 90 120, 180, 270 and 360 days post cessation. Self-reported abstinence was verified by expired carbon monoxide at every follow-up visit. Salivary cotinine levels were monitored after nicotine gum use was discontinued. Abstinence was determined on an intent-to-treat basis, in that the participants who reported for the baseline visit and pre-quit visit received the NRT with behavioral counseling but at some subsequent time became lost to follow-up, were considered to have relapsed. Both the CO and salivary cotinine were measured at the baseline visit as well. Whether the intervention led to any harm reduction is an important issue and will be evaluated in the relevant analysis and further reported in a separate paper. The current paper focuses on the baseline risk behaviors as predictors of quitting.

### Statistical analyses

Univariable statistical methods included chi-square analyses with categorical variables, and analyses of variance plus Tukey's HSD post hoc tests for independent samples with continuous variables. Nicotine dependence, depressive symptoms, and self-efficacy were used as continuous sum scores in the analyses of variance. Length of the longest past quit attempt, although a continuous variable, was not normally distributed (skewness = 4.10). Thus, it was classified into 2 categories using a median split (120 days or less/more than 120 days). Education was classified into 2 categories (less than college graduate/college graduate or more).

As multivariable methods, survival analyses (proportional hazards models) were performed to examine the relationships among risk behaviors, smoking-related variables, and relapse. Reference cell coding was used in post hoc analyses to test pair-wise differences in abstinence rates among the subgroups [41]. The statistical significance of the pair-wise differences in the survival curves were tested by Log Rank and Cox's F tests. In order to analyze accumulation effect of multiple risk behaviors we used number of those behaviors as a three category variable (0, 1, 2 added risk behaviors). The classification into a risk behavior was based on the survival analyses for dietary behavior and alcohol consumption. The group of each behavior (diet or drinking) showing the strongest association with relapse was regarded as a risk behavior. For crude hazard ratios and for testing the interactions, the other explanatory variables were dichotomized as follows: nicotine dependence (high = FTND>5/low = FTND<5), longest quit attempt (short = ≤120 days/long = >120 days), depression (depressed = CES-D>15/non-depressed = CES-D≤15), self-efficacy (low = confidence score<2.5/high = confidence score≥2.5) and education (low =< college/high = ≥college). The cutoff points were selected because they provided mean splits, except the CES-D, where the cutoff point was based on earlier literature [42]. First, we computed the unadjusted survival models for diet, alcohol use and accumulation of risk behaviors (0, 1, 2), as well as for nicotine dependence, longest quit attempt, depressive symptoms, self-efficacy and education separately (crude hazard ratios). Second, we computed the adjusted models for diet, alcohol and accumulated behaviors, adjusting for nicotine dependence, longest quit attempt, depressive symptoms, self-efficacy and education (adjusted hazard ratios). Finally, we tested all possible interactions between each behavior (alcohol use, diet) and each of the other baseline variables.

## Results

The random assignment resulted in fairly equal number of both 'exercise' and "wellness' group participants in the additional risk behaviors groups (*p *= 0.830, χ^2 ^test). As shown in Table 2, dietary behavior was related to depression status at baseline (*p *= 0.053, analysis of variance). Those engaged in a mixed diet had higher depression scores than those with a high proportion of vegetables in their diet (*p *< 0.05, Tukey's test). Alcohol consumption was related to nicotine dependence (*p *= 0.026, analysis of variance). Those with moderate drinking had lower FTND score compared to those with no alcohol use at all (*p *< 0.05, Tukey's test). Alcohol use was also related to educational level. The proportion of women with less than college degree was lowest in the group with moderate drinking (*p *= 0.013, χ^2 ^test) (see Table 3).

**Table 2:** Socioeconomic, Smoking-related and Psycho-social Baseline Variables by Dietary Behavior

	High-fat diet		Mixed diet		High-vegetable diet		
	(n = 40)		(n = 64)		(n = 44)		
	%	n	%	n	%	n	*P *value

Education % < college degree	57.5	23	51.6	33	54.6	24	0.903 ^a^
Longest quit attempt % < 120 days	60.0	24	43.7	28	52.3	23	0.264^a^

	Mean	(SD)	Mean	(SD)	Mean	(SD)	*P *value

FTND ^b ^– score	5.3	2.3	4.5	2.6	5.4	2.4	0.142^c^
CESD^d ^– score	14.3^e, f^	9.9	16.1 f	9.8	11.4 ^e^	9.6	0.053^c^
Self-efficacy – score	2.4	0.9	2.5	0.9	2.4	0.8	0.950^c^

^a ^χ^2 ^test

^b ^Fagerstrom Test for Nicotine Dependence

^c ^analysis of variance and Tukey's Test

^d ^Center for Epidemiological Studies-Depression

^e, f ^mixed diet > vegetable diet; p < 0.05

**Table 3:** Socio-economic, Smoking-related and Psycho-social Baseline Variables by Alcohol Use

	0 drinks/ week (n = 43)		1–7 drinks/ week (n = 72)		>7 drinks/ week (n = 32)		
	%	n	%	n	%	n	*P *value

Education % < college degree	75.0		41.7		53.1		0.013^a^
Longest quit attempt % < 120 days	43.2	19	51.4	37	59.4	13	0.373 ^a^

Baseline variable	Mean	(SD)	Mean	(SD)	Mean	(SD)	*P *value

FTND^b ^– score	5.8^d^	2.4	4.5^e^	2.2	4.9 ^d, e^	2.8	0.026^c^
CESD^f ^– score	12.5	9.7	14.7	8.8	15.7	12.5	0.344^c^
Self-efficacy – score	2.5	0.8	2.5	0.9	2.3	0.9	0.623^c^

^a ^χ^2 ^test

^b ^Fagerstrom Test for Nicotine Dependence

^c ^analysis of variance and Tukey's Test

^d, e ^No alcohol > 1–7 drinks/week; *p *< 0.05,

^f ^Center for Epidemiological Studies-Depression

**Figure 2:**
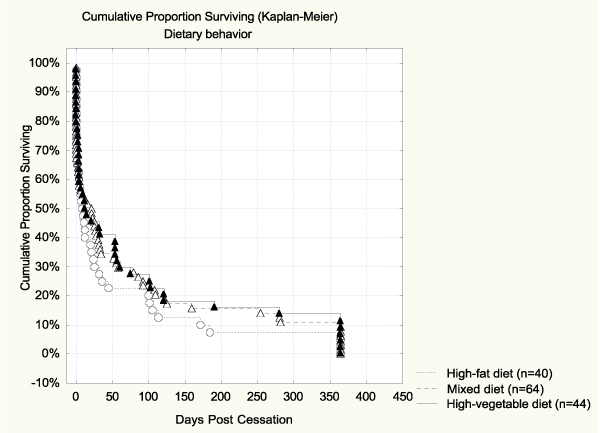
**Percent abstinent during 365 days post-cessation by diet**. No significant differences were observed.

**Figure 3:**
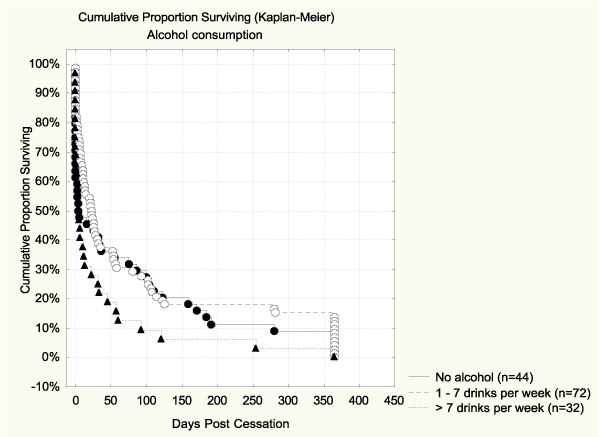
**Percent abstinent during 365 days post-cessation by alcohol consumption**. Difference between the curve of moderate drinkers and the one of high consumers was significant (*p *= 0.02 Log Rank test; *p *= 0.01 Cox F-test).

Based on unadjusted survival analysis, the quality of diet alone did not predict abstinence (see Table 4). The Hazard ratio for high-fat diet vs. high-vegetable diet was 1.27 (CI95% 0.83–1.95), but the association was not significant. The survival curve for diet is shown in Figure 2. Alcohol use alone was a significant predictor of cessation outcome. Those who consumed more than 7 drinks per week were more likely to relapse than those who used alcohol moderately (Hazard ratio = 1.62, CI95% 1.06–2.48). Those with no alcohol use showed a marginally higher risk for relapse (Hazard ratio = 1.17, CI95% 0.80–1.70). The survival curve for alcohol is shown in Figure 3, where the difference between the curve of moderate drinkers and the one of high consumers was significant (*p *= 0.020 Log Rank test; *p *= 0.01 Cox F-test).

**Table 4:** Survival Analyses of Likelihood of Relapse: Crude Hazard Ratios (n = 146)

	Hazard Ratio	95% CI^a^	χ^2^	*P *value
Diet				
High-vegetable	1.00			
Mixed	1.08	0.73–1.58	0.14	0.712
High-fat	1.27	0.83–1.96	1.20	0.272
				
Alcohol				
No alcohol	1.17	0.80 – 1.70	0.64	0.422
1–7 drinks/week	1.00			
>7 drinks/week	1.62	1.06 – 2.48	5.02	0.025
				
Accumulation of risk behaviors				
No risk behaviors	1.00			
>7 drinks/week or high-fat diet	1.30	0.93 – 1.83	2.34	0.126
>7 drinks/week and high-fat diet	2.56	1.16 – 5.62	5.44	0.020
				
Nicotine dependence				
Low (FTND≤5)	1.00			
High (FTND>5)^b^	1.05	0.76 – 1.46	0.09	0.760
				
Longest quit attempt				
Long (≤120 days)	1.00			
Short (<120 days)	1.25	0.91–1.73	1.86	0.172
				
Baseline depression				
Non-depressed (CESD≤15)	1.00			
Depressed (CESD>15)^c^	1.02	0.73–1.42	0.01	0.919
				
Self-efficacy				
High (confidence score≥2.5)	1.00			
Low (confidence score<2.5)	1.07	0.77–1.47	0.14	0.706
				
Education				
High (≥college degree)	1.00			
Low (<college degree)	1.12	0.81–1.55	0.46	0.499

^a ^CI = Confidence Interval

^b ^FTND = Fagerstrom Test for Nicotine Dependence

^c ^CESD = Center for Epidemiological Studies-Depression

**Figure 4:**
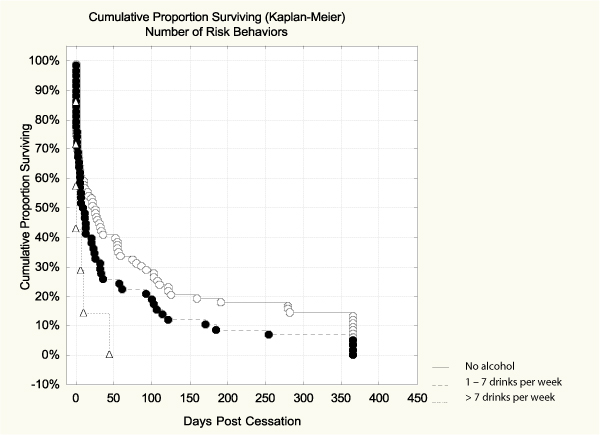
**Percent abstinent during 365 days post-cessation by accumulation of risk behaviors**. The difference between the curve of two risks and the one of no risks was significant (*p *= 0.001 Cox F-test).

The accumulation of risk behaviors was analyzed in three groups as follows: no risk behaviors (n = 83), i.e. no heavy alcohol use and no high-fat diet, one risk behavior (n = 58), i.e. heavy alcohol use without high-fat diet (n = 25) or high-fat diet without heavy alcohol use (n = 33), and two risk behaviors (n = 7), i.e. heavy drinking and high-fat diet. When analyzed by this accumulation of added risk behaviors, those who consumed >7 drinks per week and had high-fat diet, showed a significantly higher risk for relapse than those without those added risk behaviors (Hazard ratio = 2.56, CI95% 1.16–5.62). The survival curves for accumulation effect and numbers of participants in each group are shown in Figure 4, where the difference between the curve of 2 risks and the one of no risks was significant (*p *= 0.001 Cox F-test).

None of the other baseline variables studied had any significant main effect on abstinence. When adjusting for the other variables (Table 5), the associations of alcohol alone (1.62; 1.06–2.48) and alcohol accumulated with high-fat (2.56; 1.16–5.62) remained significant. Finally, we tested the interactions of heavy alcohol use and high-fat diet with all other baseline variables. Only one significant interaction was found, i.e. between baseline depressive symptoms and dietary behavior (*p*= 0.019). When adjusting for other baseline variables the quitters with high CESD scores were three times as likely to relapse (Hazard ratio = 3.02, CI95% 1.20–7.57) in comparison to those with high vegetable diet and low depression score (Table 6). The pair-wise comparisons between each subgroup based on reference cell coding are shown in Table 7. The survival curves of subgroups by depressive symptoms and diet group are shown in Figure 5a and 5b. Based on both pair-wise comparison tests, the survival of those with high depression scores and high-fat diet was significantly poorer than among those depressed with high-vegetable diet (*p *= 0.02 Log Rank test; *p *= 0.01 Cox F test). Among the participants with low depression scores there was no significant difference by dietary behavior in abstinence.

**Table 5:** Survival Analyses of Likelihood of Relapse: Adjusted^a ^Hazard Ratios (n = 146)

	Hazard Ratio	95% CI^b^	χ^2^	*P *value
Diet				
High-vegetable	1.00			
Mixed	1.16	0.76–1.75	0.47	0.490
High-fat	1.27	0.81–1.99	1.11	0.292
				
Alcohol				
No alcohol	1.10	0.71–1.69	0.18	0.669
1–7 drinks/week	1.00			
>7 drinks/week	1.62	1.05–2.50	4.66	0.031
				
Accumulation of risk behaviors				
No risk behaviors	1.00			
>7 drinks/week or high-fat diet	1.30	0.92–1.84	2.22	0.136
>7 drinks/week and high-fat diet	2.57	1.08–6.09	4.59	0.032

^a ^Adjusted for nicotine dependence, longest quit attempt, baseline depression, self-efficacy, and education

^b ^CI = Confidence Interval

**Table 6:** Survival Analyses of Likelihood of Relapse: Interaction Model with Adjusted ^a ^Hazard Ratios (n = 146)

	Hazard Ratio	95% CI^b^	χ^2^	*P *value
Diet				
High-vegetable	1.00			
Mixed	1.04	0.62–1.75	0.03	0.873
High-fat	0.85	0.48–1.50	0.31	0.579
Baseline depression				
Non-depressed (CESD≤15)	1.00			
Depressed (CESD>15)^c^	0.72	0.38–1.37	1.01	0.314
Interaction: Diet × Depression				
High-veg. diet × Non-depressed	1.00			
Mixed diet × Depressed	1.37	0.61–3.07	0.58	0.447
High-fat diet × Depressed	3.02	1.20–7.57	5.52	0.019

^a ^adjusted for nicotine dependence, longest quit attempt, self-efficacy, education

^b ^CI = Confidence Interval

^c ^CESD = Center for Epidemiological Studies-Depression

**Table 7:** Pair-wise Comparisons between Groups by Depression and Diet^a^

	χ^2^	*P *value^b^
Depressed+No High-fat Diet *vs*. Depressed+High-fat diet	6.48	0.011
Non-Depressed+No High-fat Diet *vs*. Non-Depressed+High-fat diet	0.31	0.579
Non-Depressed+No High-fat Diet *vs*. Depressed+High-fat diet	3.68	0.055
Non-Depressed+No High-fat Diet *vs*. Depressed+ No High-fat Diet	1.01	0.314
Non-Depressed+High-fat diet *vs*. Depressed+High-fat diet	0.91	0.339
Non-Depressed+High-fat diet *vs*. Depressed+No High-fat Diet	0.24	0.623

^a ^adjusted for nicotine dependence, longest past quit attempt, self-efficacy, education

^b ^based on reference cell coding

**Figure 5a:**
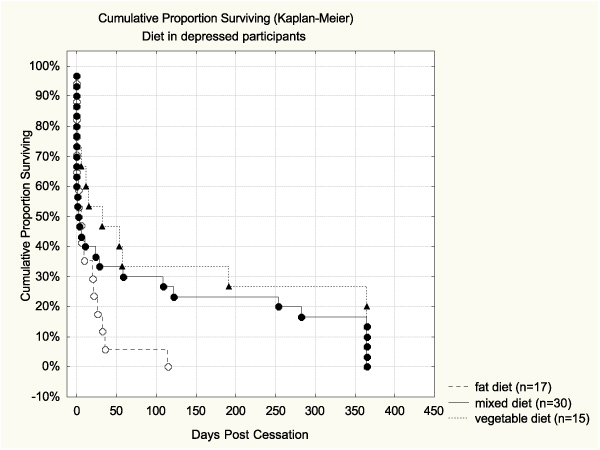
**Percent abstinent during 365 days post-cessation by diet group in depressed participants**. Abstinence of those with high depression scores and high-fat diet was significantly poorer than among those depressed with high-vegetable diet (*p *= 0.02 Log Rank test; *p *= 0.01 Cox F test).

**Figure 5b:**
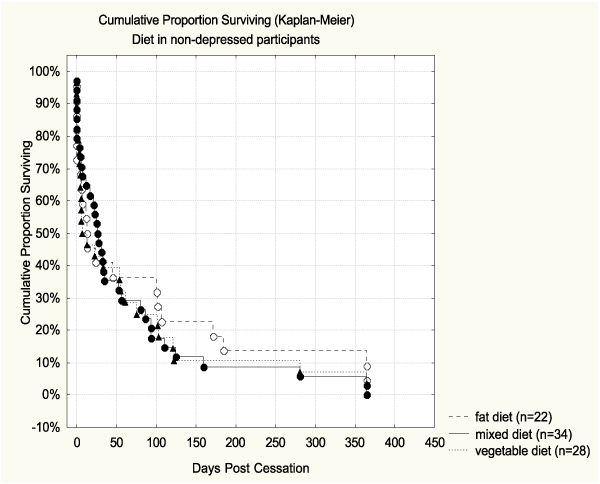
**Percent abstinent during 365 days post-cessation by diet group in non-depressed participants**. Among the participants with low depression scores no significant differences by dietary behavior were observed.

## Discussion

This study suggested that added CVD risk behaviors such as high alcohol consumption predict poorer cessation outcome in a quit attempt. The accumulation of added risk behaviors – although reflecting a relatively small number of participants – also predicted lower abstinence. Dietary behavior alone was not related to cessation outcome. However, being engaged in high-fat diet seemed to interact with depression, suggesting that depressed women engaging in high-fat diet are more likely to relapse in their quit attempt.

Sherwood et al. [28] found that the baseline multiple health risk behaviors were associated with higher Fagerstrom Nicotine Dependence (FTND) scores and lower self-efficacy from refraining from smoking. Our results partly support these results as our data showed a marginally significant association between alcohol use and FTND. However, in the present analysis self-efficacy, measured as baseline confidence to quit smoking, was not associated with engaging in additional risk behaviors. Studies among a population sample and among hospitalized smokers have found that alcohol use had stronger associations with smoking-related variables, such as nicotine dependence, than with variables related to smoking cessation motivation [43,44].

Regarding smoking cessation outcome, we found that multiple compromising health behaviors are significantly related to poor abstinence among women, whereas an earlier study [28] found a significant association among men only. Different results may be due to different health behaviors examined in these two studies; i.e. diet and alcohol use in the present analysis versus diet and physical activity in Sherwood's study [28]. Also, all our participants already had two risk behaviors; i.e. smoking and sedentary lifestyle.

Quality of the diet alone did not predict cessation outcome, but alcohol use alone was a significant predictor. Those who consumed more than seven drinks per week were significantly more likely to relapse than those with moderate drinking. The hazard ratio for those who did not consume alcohol at all was elevated, too, but did not reach statistical significance. Alcohol use seems to be quite unstable as a predictor of smoking cessation. For example, a recent cross-sectional study did not show significant associations between alcohol use and smoking cessation [45]. A further challenge is the definition of alcohol consumption as CVD risk behavior. According to the J-shaped relationship of alcohol use and risk of CVD, non-use of alcohol on one hand, and more than 7 drinks per week on the other hand should be regarded as alcohol-related risk behavior for CVD [23]. This J-shaped association has been recently challenged among smokers [24]. In relation to smoking cessation, we analyzed alcohol consumption in three separate categories. Based on our results, the definition of cessation related risk behavior included high alcohol consumption only. Thus, similar J-shaped association as suggested for CVD risk could not be replicated in relation to smoking cessation.

We used nicotine dependence, length of the longest past quit attempt, depressive symptoms, self-efficacy, and education as baseline predictors to be analyzed in addition to health risk behaviors. One issue is whether there is evidence of those factors predicting abstinence. Recent studies indicate that including nicotine dependence, length of the longest past quit attempt, depression and education were relevant to analyses of cessation [46,47]. However, another recent study suggests that the effect of education disappears when adjusting for other variables, such as other health behaviors and social environment [45]. Among female smokers in our trial, any of these variables as such had neither unadjusted nor adjusted effect on abstinence. However, after controlling for all other baseline variables the relationship between accumulation of risk behavior and cessation outcome was slightly attenuated, yet remaining significant (p = 0.032). Thus, it seems that controlling for those variables was relevant.

Although dietary behavior alone was not related to smoking cessation outcome we found a significant interaction between diet and depression. Women (n = 17) who had high depression scores and high-fat diet had significantly highest risk for relapse. One could ask whether these participants' weight or weight concerns has something to do with this result. The baseline average body mass index (BMI) was only marginally higher among the high-fat group (mean = 27.4 vs. 26.2 in mixed and 25.7 in vegetable diet; p = 0.380). In subgroups by depression and diet interaction there was no significant difference. However, the weight concerns were highest in the high-fat diet group with high depression score (weight concern mean score = 14.1, SD = 5.7) in comparison to other subgroups such as the high-fat group with low depression score (mean = 11.0, SD = 5.8), the depressed (mean = 12.3, SD = 5.3) and the non-depressed with mixed or vegetable diet (mean = 10.3, SD = 5.7). These differences were almost significant (*p *= 0.070). We also carried out a further interaction model adjusted for weight concerns at baseline, but this did not radically change the results shown in table 6. It looks like weight concerns may be an issue to be analyzed in larger samples in relation to dietary behavior and depression.

Interaction between a health risk behavior and depressive symptoms in association with smoking cessation has been reported earlier among smokers in outpatient alcoholic treatment [48]. In this particular study, the interaction suggested that greater number of days since last drink was associated with greater readiness to quit, being significant only among patients with low depression scores. In the present study, alcohol use had a direct effect only, but no significant interaction with depression.

Interestingly, the depressed women who did not use high-fat diet were relatively successful in this study. According to the pair-wise comparisons, these women were not significantly different from any of the non-depressed women. The only significantly less successful group was the depressed women with high-fat diet. Partly this may be a surprising result, as usually depressive symptoms at baseline predict low success in smoking cessation. It is possible that the relatively low weight concerns of these depressed women – with a healthier diet – have contributed to this result. This notion, however, needs to be investigated in a larger sample. One explanation may be that in our trial all subjects received NRT. In an earlier study NRT was particularly beneficial for depressed quitters [42]. It looks like NRT could assist depressed smokers to reduce the gap to the non-depressed ones measured at baseline. This result could not be explained by higher nicotine dependence because after controlling for FTND the results remained the same.

Every woman in our study already had at least 2 main CVD risk factors, i.e. smoking [49] and a sedentary lifestyle [50]. In addition, some 40% (n = 65) had 1–2 additional risk factors, i.e. unfavorable diet and/or alcohol use [22,23]. Further, 30% had at baseline high level of depressive symptoms, which is also a risk factor for development of CVD [51]. Hence, our results raise a further question whether multiple risk behavior interventions should be combined to reduce the total burden of their CVD risk factors. It has been suggested that change in one risk behavior may relate to change in another. For example, the cognitive mechanisms associated with changes in smoking behavior are related to the cognitive variables which have been shown to predict changes in other behaviors [52]. The readiness to change multiple risk behaviors was studied among nicotine and alcohol dependent outpatients [53]. Patients reported higher confidence to abstain from alcohol than from cigarettes. Those with high motivation for changing alcohol use and low motivation to quit smoking remained longer in the program, whereas those with high motivation for changing both behaviors dropped out early. It seems that, in spite of readiness to change dual-dependency behaviors, actual quitting both simultaneously may prove difficult. Smoking cessation in dual-dependence programs may be less successful than in interventions targeting smoking only. However, in terms of total cardiovascular risk profile, some combined interventions may produce higher public health impact. For example, smoking cessation together with exercise or dietary intervention may have significant combined effects, although the absolute quit rates would not be highest.

Regarding limitations of the study, our sample was relatively small and selected. Our participants represent smoking women who are willing to quit smoking and who have sedentary lifestyle. Thus, our results are suggestive and further studies with larger samples are needed to confirm the significance of multiple CVD risk behaviors and their interactions with other smoking related variables. Further, although the participants had a sedentary lifestyle at baseline, this may not be true for all of them once they commenced the study. Specifically, the exercise intervention, if optimally followed, would make these participants more active during their quit process. Of the three groups in the study both exercise and wellness had similar one year abstinence rates. However, the mechanisms by which this was achieved are not clear. In regard to adopting exercise into their sedentary lifestyles we analyzed the self-reported frequency of exercise behavior at the baseline and at the end of treatment at 16 weeks after quitting. Based on the data available at 16 weeks, the three risk groups did not differ in changes from the baseline in exercise behavior (F = .608 (2) *p *= 0.549), and hence suggest that increased exercise does not explain the observed differences. It has to be noted though that data on 16 week exercise status was only available for 4 participants in the risk group of 2 risk behaviors. Further, the relapse to smoking and subsequent drop out was rapid, and thus, there was a minimal time for major changes in health behaviors to occur. Finally, as an additional limitation of the study, we have to note that both added risk behaviors were measured by self-reports. It is possible that there has been some under-reporting of alcohol use and recall bias associated with one-week retrospective dietary reports versus food diaries. Thus, this possible bias may have caused slight dilution of these behaviors' effects on the results.

## Competing interests

The authors declare that they have no competing interests.
